# Supplementary material for: A Subpathway and Target Gene Cluster-Based Approach Uncovers lncRNAs Associated with Human Primordial Follicle Activation
Source: Int J Mol Sci. 2023 Jun 23;24(13):10525. doi: 10.3390/ijms241310525 (PMC10342156; doi:10.3390/ijms241310525)
Supplement: Supplementary file 1 [file ijms-24-10525-s001.zip › Supplementary Figures.pdf]

Supplementary Material
for

A Subpathway and Target Gene Cluster-Based Approach Uncovers lncRNAs Associated with Human Primordial Follicle Activation

Li Zhang †, Jiyuan Zou †, Zhihao Wang and Lin Li *

Guangdong Provincial Key Laboratory of Proteomics, Department of Pathophysiology, School of Basic Medical Sciences, Southern Medical University, Guangzhou 510515, China; bio_minizhang@outlook.com (L.Z.); zouzouzoujiyuan@163.com (J.Z.); vinod_wang@163.com (Z.W.)
* Correspondence: lilin2019@i.smu.edu.cn; Tel./Fax: +86-20-61648843
† These authors contributed equally to this work.

This PDF file includes:
Figures S1 to S3
Legend for Tables S1–S4

Supplementary Table:
Table S1. Differential expressed lncRNA in follicles (n = 65) and granulosa cells (n = 22) during PFA.
Table S2. LncRNA ranks in follicles during PFA.
Table S3. 725 KEGG subpathways identified by k-clique algorithm.
Table S4. Premature ovarian insufficiency (POI)-related genes (n = 127).

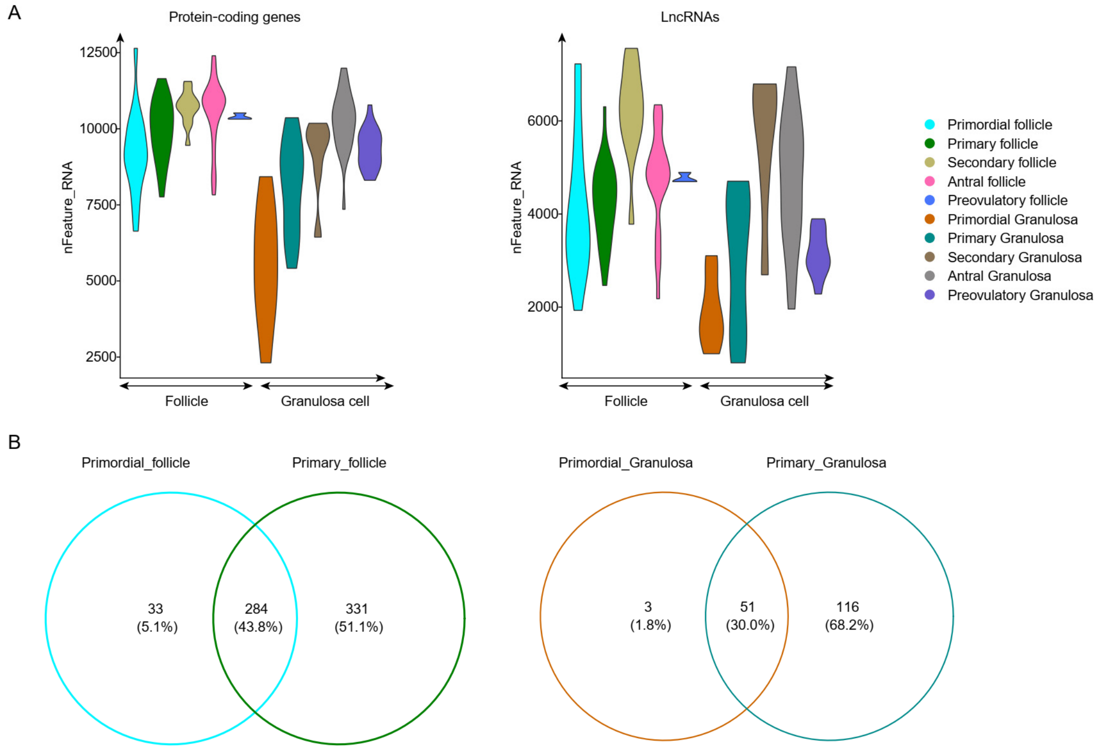

Figure S1. Distribution of expressed protein coding genes and lncRNAs during follicle development. (A) Violin plots show the number of expressed protein-coding genes (left) and lncRNAs in follicles and granulosa cells from primordial to preovulatory stage. (B) Venn diagram analysis of expressed lncRNAs in follicles (left) and granulosa cells (right) during PFA.

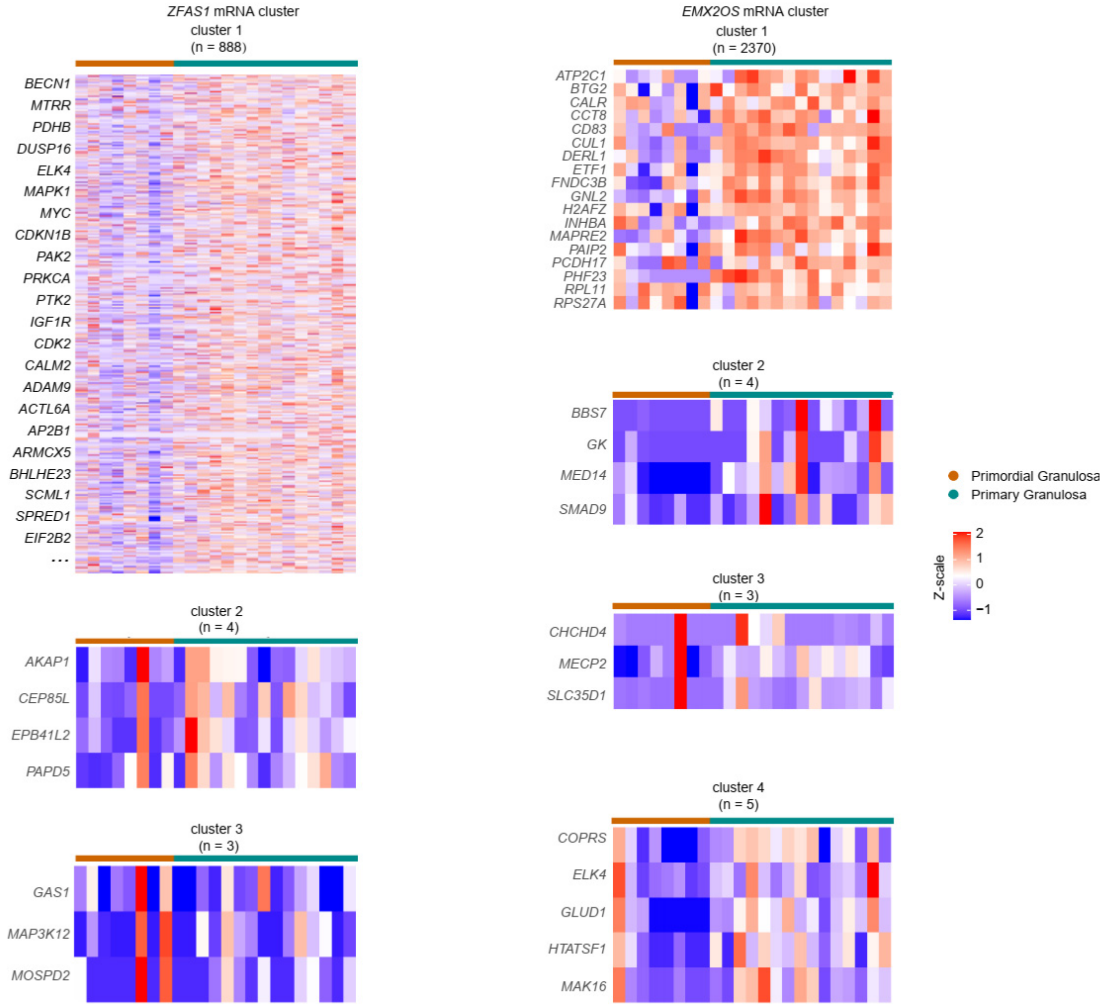

Figure S2. Heat maps of cluster genes of *ZFAS1* and *EMX2OS* in primordial and primary granulosa cells.

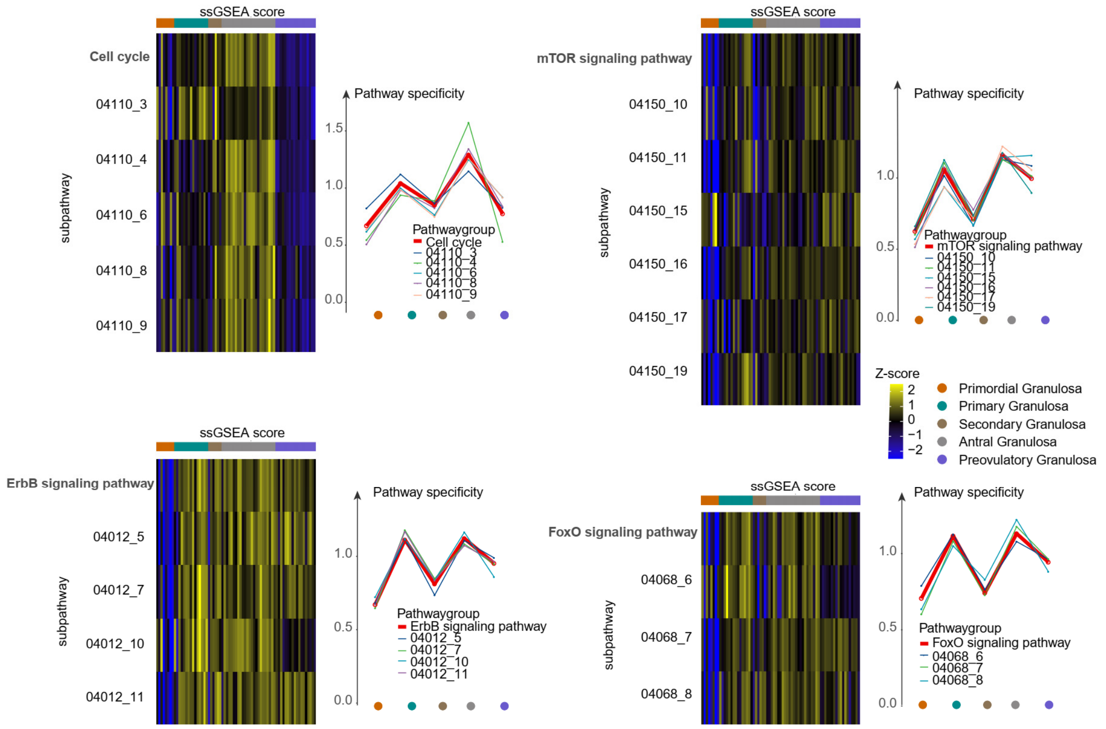

Figure S3. The difference between "Cell cycle", "mTOR signaling pathway", "ErbB signaling pathway", "FoxO signaling pathway" and their corresponding subpathways in granulosa cells from primordial to preovulatory stage. The left heat map showing the ssGSEA score between pathway and subpathways. The right line chart indicates the dynamic change of pathway specificity, red line represents the entire pathway, whereas other color lines represent corresponding subpathways.
